# Impact Localization in Complex Cylindrical Shell Structures Based on the Time-Reversal Virtual Focusing Triangulation Method

**DOI:** 10.3390/s24165185

**Published:** 2024-08-11

**Authors:** Xiufeng Huang, Rongwu Xu, Wenjing Yu, Shiji Wu

**Affiliations:** 1 Laboratory of Vibration and Noise, Naval University of Engineering, Wuhan 430033, China; 1820102@nue.edu.cn (X.H.);; 2 National Key Laboratory of Vibration and Noise on Ship, Naval University of Engineering, Wuhan 430033, China

**Keywords:** large-scale compartmental structures, time-reversal virtual focusing, triangulation localization, impact localization

## Abstract

In addressing the challenging issue of impact source localization for large-scale anisotropic stiffened compartmental cylindrical shell structures, this paper presents a novel impact localization method. The method is based on a time-reversal virtual focusing triangulation approach and does not rely on prior knowledge of the structure or specific measurements of wave velocity. By employing energy power filtering to select key sensors, wavelet packet decomposition is utilized to extract narrowband Lamb wave signals, which are then synthesized. Further enhancement of signal recognition is achieved through time-reversal amplification techniques. Experimental results demonstrate that under non-motorized operating conditions, this method achieves an average error of 0.89 m. Under motorized operating conditions, the average error is 1.12 m. Although the presence of background noise leads to an increase in error, the overall localization performance is superior to traditional triangulation methods. Additionally, selecting the top three sensors in terms of energy power ranking can more accurately record impact response.

## 1. Introduction

As the complexity of modern engineering structures increases and the emphasis on structural safety grows, structural health monitoring has become a research hotspot in fields such as ocean engineering, civil engineering, and mechanical engineering. Structural impact monitoring technology is an integral part of the structural health monitoring field, with its primary objective being to accurately identify the location of abnormal impacts on engineering structures. Especially in the course of a ship’s voyage, sudden failures such as accidental loosening of hull components are inevitable. Therefore, it is very important to quickly and accurately determine the source of these impact loads and remove faults in time to extend the service life of the ship. By deploying accelerometers and other sensors on the structure to capture impact response signals and combining modern signal-processing techniques with specific algorithms, impact localization can be achieved [1,2,3,4]. Researchers both domestically and internationally have proposed a multitude of structural impact localization methods. Representative methods include geometric methods [5], reference database methods [6], correlation coefficient methods [7], machine learning methods [8], and time-reversal methods [9].

Geometric methods are straightforward and have been applied since the early stages of impact localization research. Typically, these methods establish a set of nonlinear equations by extracting signal arrival times and leveraging geometric relationships between sensors and impact location, ultimately solving for corresponding location results. Common techniques include triangulation and quadrilateral localization methods. Yin, X. conducted research on acoustic emission source localization for two-dimensional homogeneous structures using the time difference of arrival method, successfully identifying source location [10]. Su, Y. and colleagues, aiming to enhance precision and real-time performance of impact localization in composite material structures, proposed a two-step impact localization method using triangulation measurement techniques, which can accurately calculate the elliptical region where the impact occurs [11]. Simone, A. introduced a linearized sound source localization technique composed of a cluster of four sensors [12]. Although geometric methods are simple and fast, their accuracy in complex stiffened structures significantly decreases, making it challenging to meet the demands of practical engineering applications.

The reference database method has gained attention due to its ability to achieve satisfactory localization accuracy even with a limited number of sensors. This approach involves comparing the characteristics of the current impact response signal with those stored in a database of impact response signals from all training grid points, thereby facilitating a match. During this process, the system identifies the grid point in the reference database with the highest similarity to current signal characteristics and considers its position as the predicted impact point. Peng, T. constructed a database using time differences of arrival at different sensors, calculated feature residuals of grid segmentation, achieved rapid localization of impact location, and conducted localization experiments with added noise, finding that this method still possesses good noise resistance [13]. Wu, Z., without considering the propagation speed of the impact stress wave, adopted a location vector model based on signal power and arrival time parameters to determine the location of the impact source [14]. This model, by integrating power spectral density and arrival time characteristics of the signal, constructs a location vector, thus accurately estimating the position of the impact source. However, the construction process of the aforementioned reference database method often involves high time costs. Moreover, the performance of the algorithm may be limited when faced with a limited number of training samples, leading to a decrease in accuracy.

The correlation coefficient method is also a relatively straightforward approach for impact localization. The primary concept involves calculating correlation coefficients between signals of each sensor pair. By analyzing these coefficients, it is observed that correlation coefficients between sensor pairs near the impact source are higher, thus allowing inference of the impact source’s location. Zhang, Y. and colleagues synchronized the acquisition of response signals recorded by sensors at different positions, then calculated the mutual correlation coefficients to quantify the time delay and similarity between signals, obtaining collision impact intensity at various positions of the tested piece, forming a collision impact intensity spectrum, and then locating the impact position by identifying the maximum value in the spectrum [15]. Zhang, Y., based on piezoelectric sensing signals from impact tests on large composite wall structures, utilized a cross-correlation function impact monitoring algorithm for localization calculations, successfully identifying corresponding impact locations [16]. Although the correlation coefficient method has the advantage of being simple, intuitive, and not requiring complex mathematical models, it also has limitations. For instance, it may not be accurate enough for nonlinear or non-uniformly propagating impact waves. Additionally, if sensors are too close to each other, signal overlap may lead to inaccurate correlation coefficient analysis.

With the continuous advancement of artificial intelligence technology, machine learning algorithms, particularly deep learning techniques, have begun to be applied to impact localization problems due to their adaptability in handling complex wavefield structures. These algorithms typically treat impact localization as either a regression or classification problem. Under the regression framework, key features of impact response signals are used as input variables, and the network is trained to predict the coordinate position of the impact source. In the classification framework, the structure is divided into multiple small grid areas, each corresponding to a category label. By learning the correspondence between signal features and these areas, precise classification of the impact occurrence region is achieved. This approach not only improves the accuracy of localization but also enhances the algorithm’s generalization ability to different structural characteristics. Huang, X. utilized a structure sound source localization method based on the backpropagation neural network. By sequentially striking the surface of a plate to excite signal sources at different locations, using the time delay difference between sensor signals as input and sound source location as output, they achieved good localization accuracy [17]. Seno, A. developed a passive sensing method based on an artificial neural network for localizing impact events in composite material plates under simulated environmental and operational conditions, demonstrating high accuracy and consistency under various impact conditions [18]. However, when applying this method to real structures with complex components, a vast prior knowledge database system needs to be established, which significantly increases time, cost, and consumption of computational resources. These issues lead to certain limitations in realizing the method’s large-scale engineering applications.

Time-reversal focusing method is a signal processing technique based on wave theory. The fundamental concept is that when excitation signals propagate through a structural medium, they are scattered and attenuated due to the inhomogeneity of this medium. However, when these scattered signals propagate again in the opposite direction of the original path, they focus at the location of the signal source, forming a high-resolution focal point. By measuring and analyzing the signal characteristics of this focal point, structural sound source localization can be achieved. Li, Q. utilized time-reversal theory to enhance acoustic emission signals in combination with numerical simulation finite element analysis software and established a planar model to simulate the signal emission and propagation process of the acoustic emission source, ultimately achieving high-precision sound source localization [19]. Zhao, W., based on Lamb wave propagation theory and Morlet wavelet transformation, combined with thin plate impact simulation, established a mathematical model for the localization of abnormal noise sources and proposed a method for localizing rattle noise sources of car doors, ultimately achieving the localization of the signal source [20]. However, the aforementioned time-reversal focusing methods either require measurement equipment to predetermine wave speeds or need to combine numerical simulation methods to simulate wave propagation in the monitoring area, which is not conducive to applications of these methods in impact monitoring of engineering structures. Deng, D. proposed a multi-frequency probabilistic imaging fusion method for impact localization on aircraft composite structures. By establishing a distributed sensing network, signals are subjected to continuous wavelet transform, and the time of arrival of signals is measured to establish a probabilistic imaging function. Effective imaging results are then evaluated for image fusion to ultimately determine impact location [21]. In addition, experimental verification of time-reversal focusing algorithms tends to be biased towards small and simple structures, with less application in large and complex structures [22,23,24,25,26,27,28].

Therefore, in response to the aforementioned challenges, this paper proposes an impact localization method based on a time-reversal virtual focusing triangulation approach. Initially, sensors are selected based on calculated energy power, with priority given to those ranked higher. Subsequently, the signal undergoes wavelet packet decomposition, and the central frequency is determined by the proportion of energy in wavelet packet coefficients at different frequency bands within the decomposition layers. Then, the signal is enhanced according to the principle of time-reversal virtual focusing, and finally, the average wave speed within the monitoring area is calculated to ascertain the impact source location using the triangulation method. This method does not require prior information about the structure, facilitating its practical implementation and significantly enhancing the efficiency of monitoring for complex engineering structures. Moreover, this method has been validated through large-scale complex cylindrical shell compartment structures, demonstrating promising application prospects.

## 2. Time-Reversal Virtual Focusing Triangulation Localization Method

### 2.1. Energy Power Calculation

When multiple sensors are deployed on the structural body, the energy power *P* of the vibration signal from the *i* sensor can be calculated using Equation (1).

(1) $$P\left(i\right)=\frac{1}{N}\sum_{\tau =0}^{t}s_{i}{\left(\tau \right)}^{2}$$

In the equation, *s_i_*(*τ*) denotes the vibration signal of the *i* sensor, and *N* represents the length of the vibration signal data. We selected sensors with higher energy power rankings as data inputs.

### 2.2. Signal Wavelet Packet Decomposition

When an excitation is applied to a structure, it generates an impact response signal, which is typically manifested in the form of Lamb waves [29]. The dispersion characteristics of Lamb waves result in a rich spectrum of frequency components within the original signal, leading to a pronounced dispersion effect. Therefore, advanced signal processing techniques are required to effectively extract useful information from the signal and reduce the impact of dispersion effects. Wavelet analysis, as a reliable time–frequency analysis tool, can effectively overcome the limitations of traditional short-time Fourier transform in frequency resolution, providing an accurate description of the signal’s local characteristics in both the time and frequency domains. Wavelet packet decomposition, as an extension of wavelet analysis, is based on the core concept of concentrating information energy processing to delve into the details of the signal to explore the inherent ordered structure, thereby extracting regular features from the signal. This method achieves a more refined analysis of the signal by performing a detailed multilevel division of frequency bands and further decomposing the high-frequency components that were not further refined in wavelet analysis. Wavelet packet decomposition can adaptively select frequency bands that match the specific characteristics of the signal, optimizing time–frequency resolution, thus providing a more efficient means for signal processing. Based on the characteristics of wavelet decomposition, researchers can use this technology for filtering impact response signals to remove unnecessary components and retain key information. This approach not only improves the accuracy of signal processing but also provides strong technical support for research in the fields of structural health monitoring and localization [30].

The mathematical expression of wavelet packet decomposition can be described through the following steps:

(1) Selection of the mother wavelet: Initially, a function that meets specific criteria is chosen, known as the mother wavelet, which will be used to generate the wavelet packet basis.

(2) Scaling and translation: The mother wavelet is subjected to scaling and translation operations. These operations can be represented by Equation (2).

(2) $$\psi_{a,b}(\tau )=\frac{1}{\sqrt{a}}\psi (\frac{\tau -b}{a})$$

ψ is the mother wavelet function. The parameter *a* is the scaling parameter, which controls the width of the wavelet. The parameter *b* is the translation parameter, which controls the position of the wavelet.

(3) Decompose signal: Signal *s*(*τ*) is convolved with the wavelet packet basis to obtain signal representations at different scales and positions. The convolution operation can be represented by Equation (3).

(3) $$W_{a,b}(s)=\int s(\tau )\psi_{a,b}(\tau )d\tau$$

The convolution result provides energy distribution of the signal at a specific scale *a* and position *b*.

(4) Multilevel decomposition: The decomposition is carried out in a multistage manner, with each stage yielding a finer frequency resolution. At each level, the signal can be divided into high-frequency and low-frequency components, and then wavelet packet decomposition is recursively applied to these two parts. In the multilevel decomposition process of wavelet packets, the signal is decomposed into approximation and detail components, which are then further decomposed, forming a tree-like structure. For approximation component *A_j_* and detail component *D_j_* at scale j, the decomposition can be represented.

(4) $$\begin{array}{l} A_{j+1}(t)=\int A_{j}(u)h(u-t)du\\ D_{j+1}(t)=\int A_{j}(u)g(u-t)du \end{array}$$

**Figure 1:**
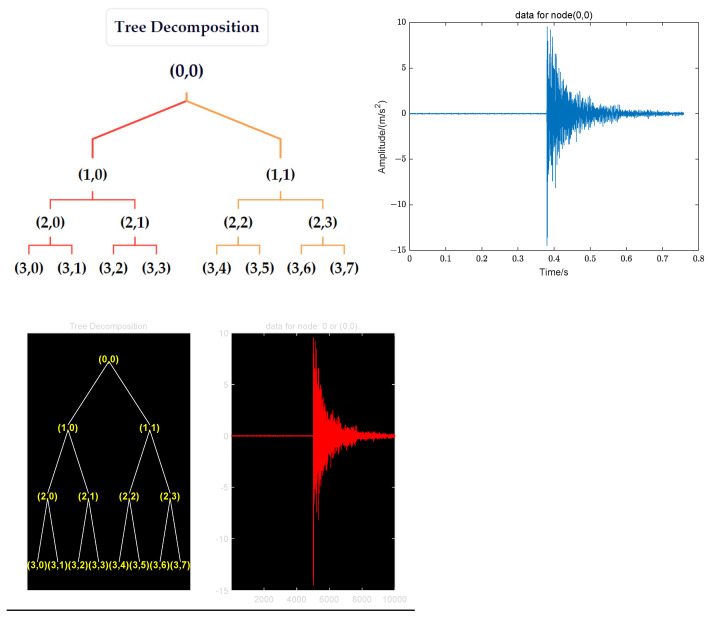
Wavelet packet decomposition schematic diagram.

Here, *h*(*t*) represents the scaling function, and *g*(*t*) denotes the wavelet function. The aforementioned procedure is repeated for each approximation and detail part until the desired level of decomposition is reached. At the *j* level, the signal is decomposed into 2*^j^* sub-bands. Wavelet packet decomposition can be illustrated using a tree diagram, known as a wavelet packet tree. Each node in the tree corresponds to a specific frequency band of the signal, with the leaf nodes representing final decomposed bands, as shown in Figure 1.

(5) Signal Reconstruction: Based on the analysis results, the signal is selectively reconstructed from sub-bands of interest. Starting from chosen sub-bands, the signal is synthesized step by step upwards until the scale of the original signal is reached. The inverse wavelet transform is then applied to convert the signal from the frequency domain back to the time domain. Finally, based on wavelet packet decomposition coefficients, an inverse transformation using wavelet basis functions can be performed to obtain the reconstructed signal.

Taking a sensor signal as an example, as shown in Figure 1. This step begins by performing a wavelet packet decomposition on the signal, yielding wavelet packet coefficients at various frequency bands of decomposition levels. Subsequently, frequencies at which the cumulative square of wavelet coefficients exceeds 80% are selected as central frequency values for the next step of wavelet transformation. Figure 1 illustrates that upon decomposing the impact response signal to three levels, there are a total of eight nodes; thus, the signal frequency can be divided into eight bands from low to high frequency, with each band’s width being *fs*/16 (where fs denotes the signal sampling rate). Consequently, the range of the first frequency band is [0, *fs*/16], the second frequency band’s range is (*fs*/16, 2*fs*/16], the third frequency band’s range is (2*fs*/16, 3*fs*/16], the fourth frequency band’s range is (3*fs*/16, 4*fs*/16], the fifth frequency band’s range is (4*fs*/16, 5*fs*/16], the sixth frequency band’s range is (5*fs*/16, 6*fs*/16], the seventh frequency band’s range is (6*fs*/16, 7*fs*/16], and the eighth frequency band’s range is (7*fs*/16, 8*fs*/16]. This paper uses frequency corresponding to cumulative energy of wavelet coefficients exceeding 80% as the central frequency and then employs a complex Morlet wavelet transform to extract the corresponding narrowband signal.

### 2.3. Time-Reversal Virtual Focusing Theory

**Figure 2:**
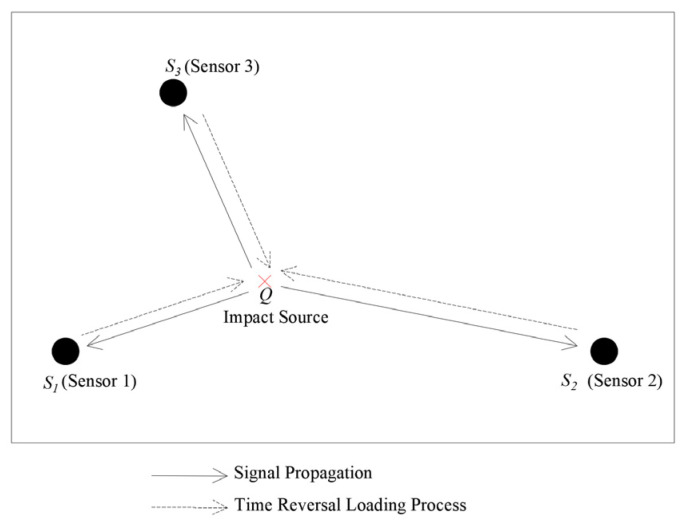
Time-reversal diagram.

Time-reversal technology is a technique based on the propagation characteristics of waves in a medium. This technique utilizes the propagation and reflection properties of waves to refocus energy at the sound source location through a time-reversal operation, thereby determining the precise location of the sound source. The specific process is as follows: After a wave emanates from a sound source, it propagates through a medium and is captured by sensors. Captured vibration signals are processed and subjected to time reversal, then reemitted along the original path in the reverse direction. This operation reverses the propagation path and the reflection effects of waves, achieving energy concentration and imaging at the sound source, as shown in Figure 2. The essence of time-reversal technology lies in the reverse processing of the temporal history of the sound source signals received by each sensor, and reemitting them into a medium. Following the order of “first to arrive, last to emit. last to arrive, first to emit”, the signals return to the sound source, reconstructing the sound source signal for precise localization [31].

When the impact source emits a signal, represented by *s*(ω) in the frequency domain, and the path transfer function from the sound source to the *i* sensor is denoted by *b* (where *i* = 1, 2, 3, 4), the signal response *z_i_*(*ω*) received by the *i* sensor can be represented by Equation (5) [32].

(5) $$z_{i}(\omega )=s(\omega )\cdot k_{i}(\omega )$$

In the equation, *ω* represents the frequency. According to signal processing theory, describing and analyzing the system’s response in the time domain using convolution is equivalent to performing multiplication in the frequency domain. Taking the complex conjugate of Equation (5) yields the time-reversed signal $z_{i}^{*}$(*ω*) of the sensor response *z_i_*(*ω*), as shown in Equation (6) below.

(6) $$z_{i}^{*}(\omega )=s^{*}(\omega )\cdot k_{i}^{*}(\omega )$$

In the equation, s*(ω) is the complex conjugate of *s*(*ω*), representing the time-reversed signal of the sound source, and $z_{i}^{*}$(*ω*) is the complex conjugate of *z_i_*(*ω*). According to the theory of reciprocity, for the same transmission path, when the positions of the sound source and the sensors are interchanged, the transfer function of the transmission path remains unchanged before and after the exchange. Therefore, by loading time-reversed signals onto $z_{i}^{*}$(*ω*), the signal at the sound source location *S*(*ω*) can be obtained, as shown in Equation (7).

(7) $$S\left(\omega \right)=\sum_{i}s^{*}\left(\omega \right)\;\cdot {k_{i}}^{*}\left(\omega \right)\cdot k_{i}\left(\omega \right)$$

In the aforementioned equation, $k_{i}^{*}$(*ω*) *·* $k_{i}$(*ω*) represents the product of the conjugate of the transfer function and itself, which results in a real number and consequently increases its magnitude. Typically, the monitoring area is equipped with multiple sensors. Thus, after applying a reverse load to each sensor, the signal will focus and superimpose at the sound source, enhancing the signal–noise ratio. This results in a significant increase in the amplitude of *S*(*ω*) compared to *s*(*ω*).

Time-reversal techniques can be categorized into two primary methodologies for specific applications: direct focusing localization and virtual focusing localization [33]. The direct focusing localization approach involves the deployment of multiple sensors around the monitoring area. These sensors, upon receiving signals emitted from a sound source, promptly apply reverse signals, thereby achieving signal focusing and enhancement at the location of the sound source. However, there are significant challenges in the practical application of this method. Firstly, the manufacturing cost of such sensors is relatively high. Secondly, to achieve direct focusing at the sound source, the sensors must be capable of both receiving and emitting signals simultaneously, the function that traditional passive monitoring sensors do not possess. Consequently, the direct focusing localization method is difficult to implement in practice, and these shortcomings limit its feasibility. An alternative approach is to employ a digital signal processing technique to substitute for direct focusing localization, thereby achieving virtual signal enhancement. This method is more feasible. Therefore, this paper adopts a digital signal processing method to achieve virtual signal enhancement. When signals received by each sensor are loaded in reverse, they will undergo secondary scattering at the sound source and subsequently be received again by sensors. This process can be specifically represented by the following Formula (8):

(8) $$Z_{j}\left(\omega \right)=S\left(\omega \right)\cdot k_{j}\left(\omega \right)=\sum_{j}s^{*}\left(\omega \right)\cdot {k_{i}}^{*}(\omega )\cdot k_{i}\left(\omega \right)\cdot k_{j}\left(\omega \right)$$

In the equation, *k_i_*(*ω*) and *k_j_*(*ω*) represent transfer functions of reception paths for the *i* sensor and *j* sensor, respectively. However, transfer functions for different sensors are not known and are prone to change with the variation of paths. Thus, further manipulation of Equation (8) is necessary. This can be accomplished by simultaneously multiplying both sides of Equation (9) by *s*(*ω*)·*s*(*ω*). The signal from sensors on the left side of the equation can be denoted by *Z*^′^*_j_*(*ω*). After this processing, the equation can be rearranged to yield Equation (9).

(9) $$\begin{array}{l} {Z^{\prime }}_{j}\left(\omega \right)=S\left(\omega \right)\cdot k_{j}\left(\omega \right)\cdot s\left(\omega \right)\cdot s\left(\omega \right)=\sum_{i}s^{*}\left(\omega \right)\cdot {k_{i}}^{*}(\omega )\cdot k_{i}\left(\omega \right)\cdot k_{j}\left(\omega \right)\cdot s\left(\omega \right)\cdot s\left(\omega \right)=\\ \sum_{i}(s^{*}\left(\omega \right)\cdot {k_{i}}^{*}(\omega ))\cdot (s\left(\omega \right)\cdot k_{i}\left(\omega \right))\cdot (s\left(\omega \right)\cdot k_{j}\left(\omega \right))=\sum_{i}{z_{i}}^{*}\left(\omega \right)\cdot z_{i}\left(\omega \right)\cdot z_{j}\left(\omega \right) \end{array}$$

Equation (9) can be interpreted as a signal excited by the sound source *s*(*ω*) · *s*(*ω*) · s*(*ω*) and received by the *j* sensor (where $k_{i}$(*ω*) and $k_{i}^{*}$(*ω*) are complex conjugates of each other and their product is a real number). Additionally, in Equation (9), $z_{i}$(*ω*) and $z_{i}^{*}$(*ω*) represent the sound source signal received by the *i* sensor and its conjugate, respectively, while *z_i_*(*ω*) denotes the sound source signal received by the *j* sensor. Since the sound source signal *s*(*ω*) shares the same frequency characteristics with *s*(*ω*) · *s*(*ω*) · s*(*ω*), it does not affect the subsequent processing. Therefore, following the aforementioned processing steps can enhance the signal energy at the sound source during monitoring, thereby improving the signal–noise ratio. $k_{i}^{*}$(*ω*) $k_{i}$(*ω*) $z_{i}^{*}$(*ω*) $z_{i}$(*ω*).

### 2.4. Triangular Positioning Method

**Figure 3:**
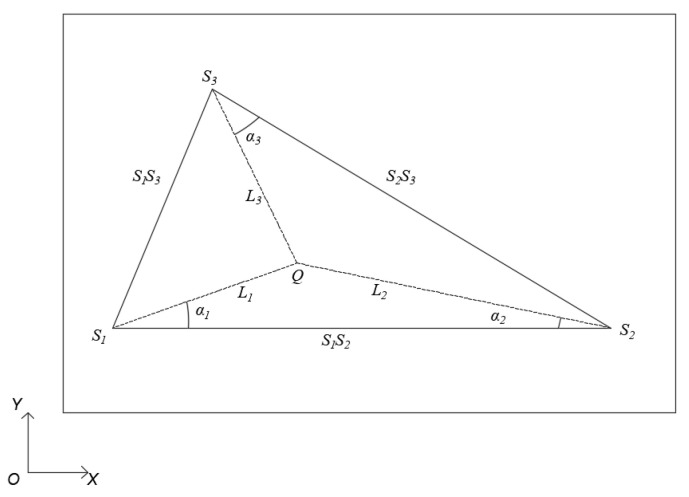
Triangular positioning method.

In the structural plane, three sensors of *S_1_*, *S_2_*, and *S_3_* are positioned to form a triangular configuration with the assumption that there is an acoustic source *Q*(x_0_, y_0_) within the triangle. Distances and angular relationships between the sensors and the sound source are depicted in Figure 3.

**Figure 4:**
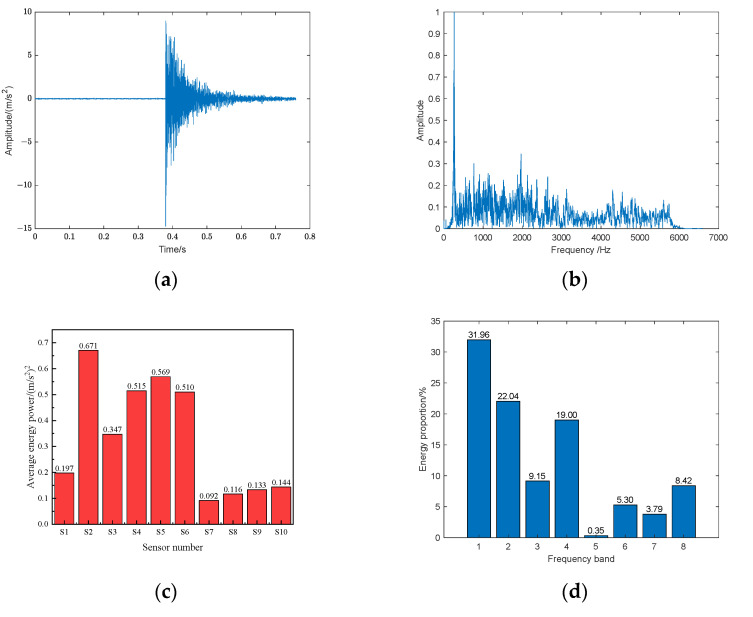

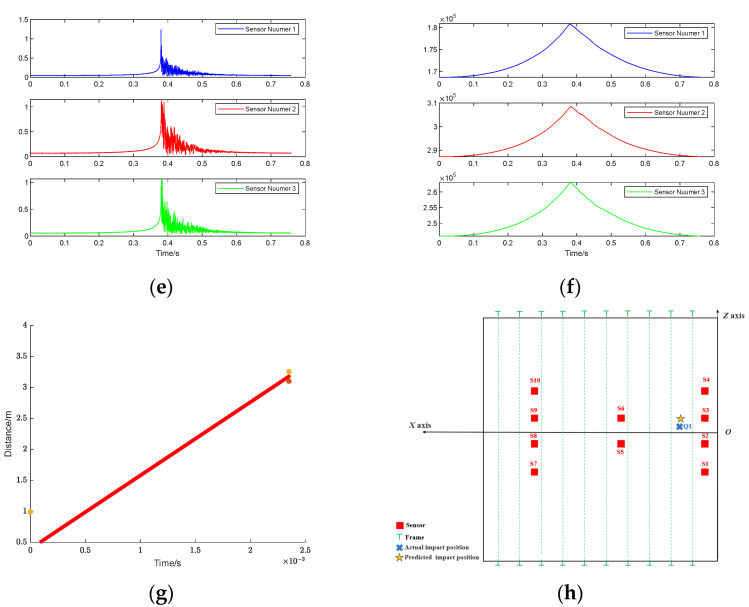
Experimental layout diagram: (**a**) time-domain signal; (**b**) spectrum signal; (**c**) energy and power calculation; (**d**) energy ratio of different frequency bands; (**e**) complex Morlet wavelet transform; (**f**) time-reversal virtual focusing processing; (**g**) average wave velocity calculation; (**h**) localization result.

Firstly, collected data signals are processed through a complex Morlet wavelet transform, yielding results shown in Figure 4e. Subsequently, signals are subjected to time-reversal focusing treatment, which results in the outcome depicted in Figure 4f. Finally, this paper selects the time axis value corresponding to the signal peak at this point as the arrival time of the wave, thereby calculating the time *t_i_* it takes for the impact response signal to propagate to each sensor. Subsequently, the time difference of arrival Δ*t_i_* between the sensor *i* and the sensor *j* can be solved. Specifics are illustrated in Equations (10) and (11).

(10) $$\Delta t_{12}=t_{1}-t_{2}=\frac{L_{1}}{c_{1}}-\frac{L_{2}}{c_{2}}$$

(11) $$\Delta t_{23}=t_{2}-t_{3}=\frac{L_{2}}{c_{2}}-\frac{L_{3}}{c_{3}}$$

In the given scenario, *L*_1_, *L*_2_, and *L*_3_ denote respective distances from the acoustic source to the sensors *S*_1_, *S*_2_, and *S*_3_. Furthermore, *c*_1_, *c*_2_, and *c*_3_ represent the wave velocities at which the sound source signal reaches the sensors *S*_1_, *S*_2_, and *S*_3_, respectively. These parameters are essential for calculating the time of arrival and the time difference of arrival, which are critical in the fields of acoustic signal processing and sensor array theory.

Based on the geometric relationship of the triangle depicted in Figure 3, the following relational Equations (12)–(15) can be established [34]:

(12) $$L_{1}\sin(\alpha_{1})=L_{2}\sin(\alpha_{2})$$

(13) $$L_{1}\cos(\alpha_{1})+L_{2}\cos(\alpha_{2})=S_{1}S_{2}$$

(14) $$L_{2}\sin(\overset{⏜}{S_{2}}-\alpha_{2})=L_{3}\sin(\alpha_{3})$$

(15) $$L_{2}\cos(\alpha_{3})+L_{3}\cos(\overset{⏜}{S_{2}}-\alpha_{2})=S_{2}S_{3}$$

In the context provided, let *S*_1_*S*_2_ represent the distance between sensor *S*_1_ and sensor *S*_2_. *S*_2_*S*_3_ represents the distance between sensor *S*_2_ and sensor *S*_3_. Consequently, leveraging Equations (12) and (13), one can derive Equations (16) and (17). Similarly, by applying Equations (14) and (15), one can calculate Equations (18) and (19).

(16) $$L_{1}=\frac{S_{1}S_{2}}{\sin(\alpha_{1})(ctg(\alpha_{1})+ctg(\alpha_{2}))}$$

(17) $$L_{2}=\frac{S_{1}S_{2}}{\sin(\alpha_{2})(ctg(\alpha_{1})+ctg(\alpha_{2}))}$$

(18) $$L_{2}=\frac{S_{2}S_{3}}{\sin(\overset{⏜}{S_{2}}-\alpha_{2})(ctg(\alpha_{3})+ctg(\overset{⏜}{S_{2}}-\alpha_{2}))}$$

(19) $$L_{3}=\frac{S_{2}S_{3}}{\sin(\alpha_{3})(ctg(\alpha_{3})+ctg(\overset{⏜}{S_{2}}-\alpha_{2}))}$$

Within the context provided, $\overset{⏜}{S_{2}}$ is the internal angle at vertex *S*_2_ of the triangle. By substituting Equations (16) and (17) into Equation (10), the Equation (20) can be derived. Similarly, by incorporating Equations (18) and (19) into Equation (11), Equation (21) can be obtained:

(20) $$\frac{1}{ctg(\alpha_{1})+ctg(\alpha_{2})}(\frac{1}{\sin(\alpha_{1})c_{1}}-\frac{1}{\sin(\alpha_{2})c_{2}})-\frac{\Delta t_{12}}{S_{1}S_{2}}=0$$

(21) $$\frac{1}{ctg(\alpha_{3})+ctg(\overset{⏜}{S_{2}}-\alpha_{2})}(\frac{1}{\sin(\overset{⏜}{S_{2}}-\alpha_{2})c_{2}}-\frac{1}{\sin(\alpha_{3})c_{3}})-\frac{\Delta t_{23}}{S_{2}S_{3}}=0$$

For anisotropic complex structures, where the wave velocity *c* varies in different directions, the average wave velocity within the region can be used as a surrogate for the wave velocity in all directions. This specific calculation method involves using the time corresponding to the peak of the signal after time-reversal virtual focusing processing as the arrival time of the stress wave at that sensor, thereby calculating the time difference of the arrival between different sensors. Finally, based on the distance between the selected sensors and time difference, the slope of the fitted line is used as an estimated wave velocity value. This average velocity is then used to replace wave velocity within the anisotropic structure region [35,36].

Consequently, Equations (20) and (21) can be transformed into forms of Equations (22) and (23) as follows:

(22) $$\frac{1}{ctg(\alpha_{1})+ctg(\alpha_{2})}(\frac{1}{\sin(\alpha_{1})\bar{c}}-\frac{1}{\sin(\alpha_{2})\bar{c}})-\frac{\Delta t_{12}}{S_{1}S_{2}}=0$$

(23) $$\frac{1}{ctg(\alpha_{3})+ctg(\overset{⏜}{S_{2}}-\alpha_{2})}(\frac{1}{\sin(\overset{⏜}{S_{2}}-\alpha_{2})\bar{c}}-\frac{1}{\sin(\alpha_{3})\bar{c}})-\frac{\Delta t_{23}}{S_{2}S_{3}}=0$$

Within this context, the internal angle $\overset{⏜}{S_{2}}$ can be determined using the law of cosines for triangles, based on relationships of the three sides *S*_1_*S*_2_, *S*_2_*S*_3_, and *S*_1_*S*_3_. This is illustrated in Equation (24) as follows:

(24) $$\cos(\overset{⏜}{S_{2}})=\frac{{\left(S_{1}S_{2}\right)}^{2}+{\left(S_{2}S_{3}\right)}^{2}-{\left(S_{1}S_{3}\right)}^{2}}{2\cdot (S_{1}S_{2})\cdot (S_{2}S_{3})}$$

Consequently, Equations (22) and (23) involve only three unknowns, specifically *α*_1_, *α*_2_, and *α*_3_. Additionally, given that Equations (17) and (18) are equivalent, the relationship among *α*_1_, *α*_2_, and *α*_3_ can be deduced, as presented in Equation (25).

(25) $$ctg(\alpha_{3})=\frac{\left(S_{2}S_{3})\sin(\alpha_{2})(ctg(\alpha_{1})+ctg(\alpha_{2})\right)}{\left(S_{1}S_{2})\sin(\overset{⏜}{S_{2}}-\alpha_{2}\right)}-ctg(\overset{⏜}{S_{2}}-\alpha_{2})$$

By integrating Equations (22), (23), and (25), the values of unknowns *α*_1_, *α*_2_, and *α*_3_ can be determined. Subsequently, using Equations (16)–(19), the values of *L*_1_, *L*_2_, and *L*_3_ can be calculated. Ultimately, this allows for computation of coordinates (*x_0_*, *y_0_*) of the acoustic source *Q*. If the acoustic source *Q* is located outside the triangular region, a simple alteration in the sign of the appropriate variable will suffice, as the aforementioned equations will still hold true [37].

### 2.5. Method Implementation Process

**Figure 5:**
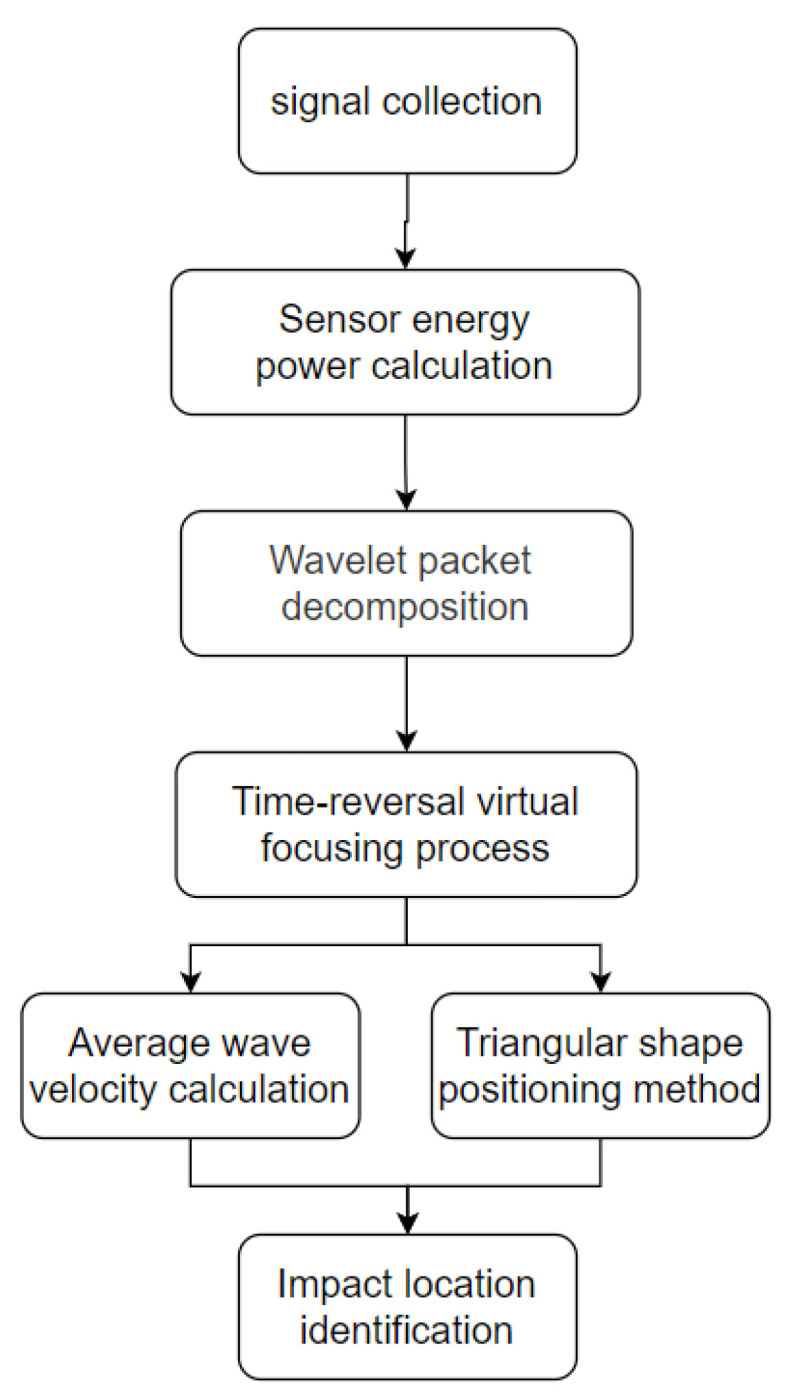
Positioning flow diagram.

This article is based on the process of time-reversal virtual focusing triangulation for localization, as depicted in Figure 5 below. Specific implementation steps are as follows:

## 3. Experimental Validation Study

### 3.1. Experiment Settings

**Figure 6:**
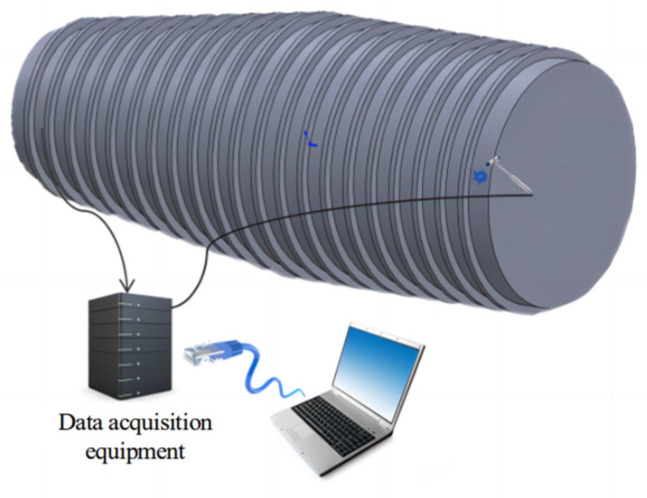
Experimental layout diagram.

The test subject is a large-scale compartment model made of Q235 material, featuring a cylindrical shell structure. Specific dimensions of the compartment are Φ3 m × 8.8 m × 0.01 m (the diameter is 3 m, the length is 8.8 m, and the thickness is 0.01 m). The entire experimental testing system consists of the compartment model, acceleration sensors, data acquisition equipment, a force hammer, and a computer, as shown in Figure 6.

**Figure 7:**
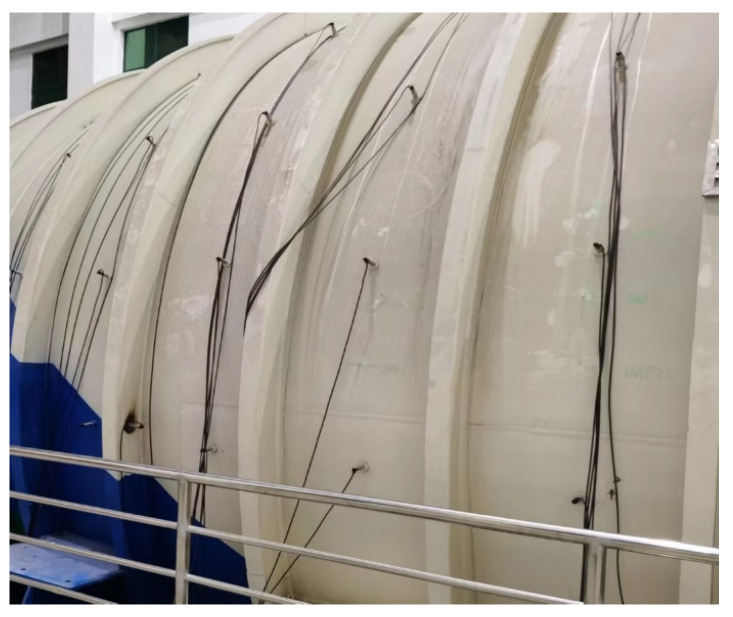
Schematic diagram of the cabin structure.

**Figure 8:**
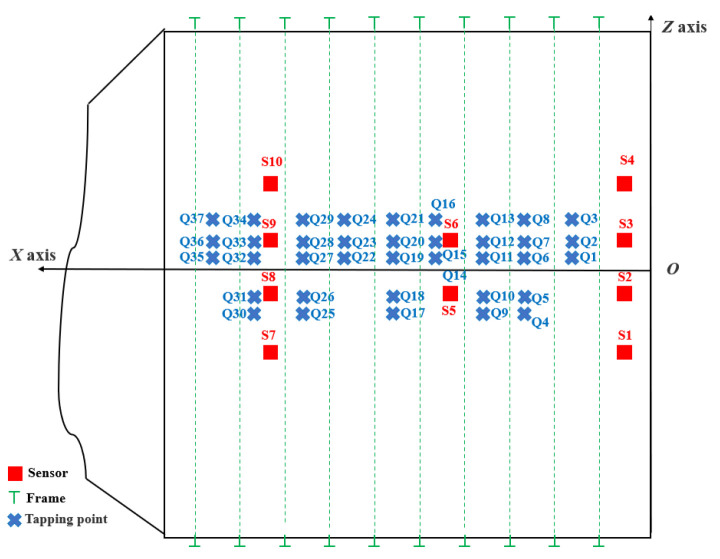
Schematic diagram of plane expansion of the cabin structure model.

To simulate sensor spacing and arrangement density in actual ship compartment structures, sparse arrangement of sensor measurement points is applied to this large-scale compartment model in Figure 7. The cabin model is equipped with motors. Acceleration sensor signal data are collected by striking corresponding positions with a hammer under two working conditions: with and without motor operating. Acceleration sensors located on the lower right side near the compartment door are designated as S1, with other sensors numbered incrementally from bottom to top and right to left. These sensors have a sampling rate of 13,183 Hz. Figure 8 shows the plane expansion diagram of the ship segment structure model. The length in the *x*-axis direction is 8.65 m, the length in the *y*-axis direction is 9.42 m, and the rib spacing is 0.80 m. Red squares represent sensor measurement points, blue “X” marks indicate striking positions, and green represents ribs.

### 3.2. Signal Processing

On the compartment structure, an impact was applied at the coordinate position (1.40 m, 0.23 m) labeled as Q1. The impact response time-domain signal captured by sensor S4 is illustrated in Figure 4a, while Figure 4b presents the normalized frequency spectrum of the impact response signal. Analysis of the spectrum reveals that the energy of the impact response signal is primarily concentrated within the 0–4 kHz frequency range, with relatively minor energy content above 4 kHz.

The average energy power values of sensors *S*_1_, *S*_2_, *S*_3_, *S*_4_, *S*_5_, *S*_6_, *S*_7_, *S*_8_, *S*_9_, and *S*_10_ are depicted in Figure 4c, with sensors *S_2_*, *S_5_*, and *S_4_* ranking as the top three in terms of energy power. A three-level wavelet packet decomposition was performed on the sensor with the highest average energy power, as shown in Figure 4d, yielding a proportion of wavelet coefficient energy across eight frequency bands. The cumulative proportion of the first four bands accounts for 82.15% (exceeding 80%). Given that the sensor’s sampling rate fs is 13,183 Hz, the frequency of the fourth band can reach 4/16*fs* (3296 Hz). Considering this comprehensively, this frequency value is extracted as the central frequency for the computation of the narrowband signal.

Utilizing the aforementioned central frequency value, a normalized complex Morlet wavelet transform is applied to signals from the top three sensors in terms of average energy power, as illustrated in Figure 4e. Subsequently, a time-reversal method is employed for signal synthesis within the structural monitoring area, followed by virtual focusing, as depicted in Figure 4f. The signal is significantly enhanced, allowing for prominent extraction and identification of signal peak values.

This paper employs the average wave speed value to represent the propagation velocity of waves within the structural domain, as depicted in Figure 4g. In Figure 4g, the three sensors with the greatest energy power are selected, and by pairing each two sensors, three pairs of sensor pairs are formed, each corresponding to the three distinct colored points in Figure 4g. Consequently, the distance difference and arrival time difference between each pair of sensor pairs can be calculated, which allows for the fitting of a straight line of distance versus time. The slope of this line is then utilized as the computed value for the average wave speed. Ultimately, positions of the impact source are calculated using the top three sensors in terms of average energy power (*S*_4_, *S*_2_, *S*_6_) in conjunction with the triangulation method, as shown in Figure 4h.

### 3.3. Results and Discussion

#### 3.3.1. Localization Results

To validate the effectiveness of the methods presented in this paper across different monitoring areas of large-scale compartment structures, the time-reversal virtual focusing triangulation localization process was repeated for 37 sound source points, from *Q*1 to *Q*37, to obtain localization results. This study employs three metrics to assess localization outcomes: localization error *e*, localization error ratio *f*, and localization accuracy *g*.

The concept of localization error *e* is defined as the difference in distance between the actual position of the acoustic source and the predicted position, as illustrated in Equation (26).

(26) $$e=\sqrt{{\left(D_{x,actual}-D_{x}\right)}^{2}+{\left(D_{y,actual}-D_{y}\right)}^{2}}$$

The meaning of localization error ratio f is the ratio of localization error *e* to the maximum dimension *L*_max_ of the monitoring area (for this structure, *L*_max_ = 12.79 m), as shown in Equation (27).

(27) $$f=\frac{e}{L_{\max}}\times 100\%$$

The definition of localization accuracy *g* is the ratio of the number of impact points *N_p_* that meet the localization error ratio requirement (error ratio less than 20%) to the total number of impact points *N*, as depicted in Equation (28).

(28) $$g=\frac{N_{p}}{N}\times 100\%$$

**Figure 9:**
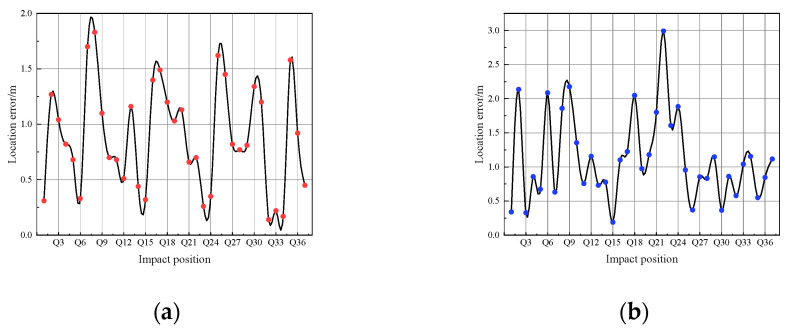
The distribution of positioning errors is illustrated: (**a**) the motor is activated; (**b**) the motor is deactivated.

The distribution of the localization error is presented in Figure 9.

In Figure 9, under the condition without motor operating, the average localization error of the large-scale compartment structure model is 0.89 m, with the maximum localization error not exceeding 2.0 m. When the motor is activated, the average localization error of the large-scale compartment structure model is 1.12 m, with the maximum localization error not exceeding 3.0 m.

When the motor is not activated, the average localization error ratio of the large-scale compartment structure model is 6.96%. When the motor is activated, the average localization error ratio increases to 8.76%. Correspondingly, the localization accuracy of the large-scale compartment structure model when the motor is not in operation is 100%, and it decreases to 97.30% with the motor activated.

Overall, while the introduction of background noise leads to an increase in localization error of the method proposed in this paper, it still maintains a relatively high level of localization accuracy and a low error ratio for such a large-scale ribbed compartment model structure. This performance is considered satisfactory for engineering applications.

#### 3.3.2. Discussion on the Top Three Sensors of Energy and Power

This paper employs the top three sensors in terms of energy power as signal inputs and implements a triangulation localization method based on time-reversal virtual focusing. However, whether sensors with the highest energy are close to the excitation source is a question worth exploring.

**Figure 10:**
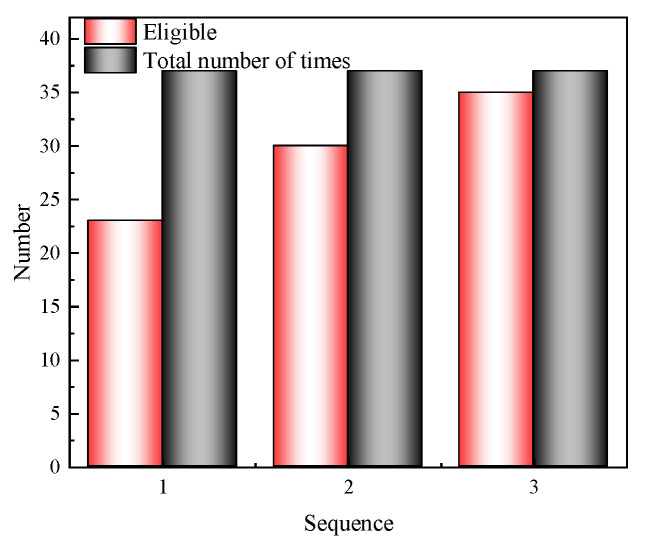
Statistical diagram.

The energy generated by impact propagates through structure in the form of waves, with its propagation path and speed depending on the physical properties and boundary conditions of the structure, such as modal characteristics, damping properties, and coupling effects. Local vibrations caused by the excitation may lead to energy concentration in specific areas of the structure or even at locations far from the excitation point, forming regions of energy concentration. This could result in sensors with the highest energy not necessarily being the closest measurement point to the excitation source. Therefore, to enhance the accuracy of analysis, it is necessary to combine experimental data from multiple impact tests for statistical analysis to reveal the regularity of energy distribution and thus determine the energy propagation pattern of sensors. Figure 10 presents the number of times sensors with the top one, top two, and top three energy power rankings, including the measurement point closest to the excitation source among all impact counts.

Figure 10 illustrates that, on the horizontal axis, the number 1 represents that, among 37 excitations, the sensor with the top energy ranking includes the measurement point closest to the excitation source for a total of 23, accounting for 62.17% of the cases. The number 2 on the horizontal axis indicates that for the sensors with the top two energy rankings, there are 30 instances where the measurement point closest to the excitation source is included, representing 81.08% of cases. The number 3 on the horizontal axis shows that for the sensors with the top three energy rankings, there are 35 instances where the measurement point closest to the excitation source is included, which is 94.59% of cases. Therefore, in this study, with rational sensor layout and effective control of testing conditions, selecting the top three sensors in terms of energy power as inputs is more likely to capture energy propagation directly caused by impact and has the highest probability of recording the most intense response.

#### 3.3.3. Comparison of Different Methods

Aiming at striking points of *Q*1–*Q*8 in the monitoring area of the large-scale cabin structure, the positioning results obtained by the proposed method are compared with those obtained by the traditional triangulation method [37], as shown in Table 1 and Table 2 below.

Comparing different localization methods, it is evident that regardless of whether the motor is off or on, the localization method presented in this paper offers higher precision and lower average localization error compared to traditional triangulation methods.

Table 1 indicates that under the condition of no motor operation, the positioning error of the method in this paper is relatively larger at the Q7 and Q8 impact points among different tapping points of Q1–Q8. This may be attributed to the fact that the Q7 and Q8 impact points are close to the upper edge of the compartment structure, where the stress wave is more likely to encounter boundaries during transmission, leading to reflection and superposition of the wave that makes calculation of arrival times less precise. However, for Q7 and Q8 positions specifically, the method proposed in this paper has a lower error compared to the traditional triangulation method. Table 2 shows that under the condition of motor operation, the positioning error of our method is relatively larger at the Q2, Q6, and Q8 impact points among the different tapping points of Q1–Q8. Specifically, at the Q8 position, the error of the method in this paper is smaller compared to the traditional triangulation method. At Q2 and Q6 positions, due to the proximity of impact points to the ribs of the compartment structure, the wave is more likely to deform during propagation, making it more difficult to accurately extract arrival times. This results in a larger positioning error for our method compared to the traditional triangulation method.

## 4. Conclusions

The method proposed in this paper, which is based on time-reversal virtual focusing triangulation for impact localization, is applicable to large-scale anisotropic ribbed cylindrical shell structures. It does not require prior knowledge of structures or specific measurements of wave velocity, facilitating monitoring and localization applications in complex engineering structures.

Utilizing energy power to identify top-ranked sensors, this method employs wavelet packet decomposition to extract the central frequency of narrowband Lamb wave signals based on the energy proportion of wavelet coefficients at different decomposition levels. These narrowband Lamb wave signals are then synthesized within the monitoring area. Envelope characteristics of synthesized signals are enhanced through time-reversal virtual focusing. Finally, impact localization is achieved by applying a triangulation method that incorporates average wave velocity.

The effectiveness of the algorithm proposed in this paper was validated on a ribbed cylindrical shell segment structure with dimensions of Φ3 m × 8.8 m × 0.01 m (the diameter is 3 m, the length is 8.8 m, and the thickness is 0.01 m). Experiments were conducted on 37 distinct impact locations with the motor both engaged and disengaged. Under the condition where the motor was disengaged, the average error was 0.89 m, the average positioning error rate was 6.96%, and the positioning accuracy was 100%. When the motor was engaged, the average error increased to 1.12 m, the average positioning error rate was 8.76%, and the positioning accuracy was 97.30%. Overall, the introduction of background noise has led to an increase in the error of positioning results.

For the compartment structure discussed in this paper, selecting sensors that rank in the top three for energy power would likely be closest to the excitation source, capturing the most intense response induced by impact.

Under various operating conditions, with and without the motor activated, 37 distinct structural locations on the compartment were subjected to impact. Compared to the traditional triangulation method, the method described in this paper exhibits lower localization error and superior localization performance.

## Figures and Tables

**Table 1:** Positioning results of various methods under the condition with the motor disengaged.

Location	Result of the Traditional Triangulation Method	Error of the Traditional Triangulation Method	Positioning Results of the Method in This Paper	Error of the Method in This Paper
*Q*1	(7.68, −0.26)	6.29	(0.86, −0.03)	0.60
*Q*2	(0.60, −0.46)	1.27	(0.60, −0.46)	1.27
*Q*3	(1.08, 0.73)	0.38	(0.60, 1.59)	1.04
*Q*4	(3.70, 0.70)	2.09	(1.95, 0.03)	0.82
*Q*5	(3.70, 0.70)	1.90	(2.70, −0.92)	0.68
*Q*6	(3.70, 0.89)	1.64	(2.08, 0.53)	0.33
*Q*7	(3.70, −0.27)	1.70	(3.70, −0.27)	1.70
*Q*8	(4.08, 0.39)	1.96	(3.70, −0.12)	1.83

**Table 2:** Positioning results of various methods under the motor-activated condition.

Location	Result of the Traditional Triangulation Method	Error of the Traditional Triangulation Method	Positioning Results of the Method in This Paper	Error of the Method in This Paper
*Q*1	(6.14, −0.26)	4.76	(1.39, 0.57)	0.34
*Q*2	(1.24, 2.09)	1.57	(0.60, −1.45)	2.14
*Q*3	(0.22, 0.84)	1.18	(1.47, 0.61)	0.33
*Q*4	(3.70, −0.14)	1.62	(2.45, 0.06)	0.86
*Q*5	(4.02, 0.83)	2.24	(2.74, −0.86)	0.67
*Q*6	(3.70, 0.75)	1.59	(3.70, 1.68)	2.09
*Q*7	(1.79, 0.12)	0.58	(1.74, 0.10)	0.63
*Q*8	(3.70, −2.96)	4.17	(2.70, −0.86)	1.86

## Data Availability

The original contributions presented in the study are included in the article material, further inquiries can be directed to the corresponding author.
